# HDAC6 is a prognostic biomarker that mediates IL-13 expression to regulate macrophage polarization through AP-1 in oral squamous cell carcinoma

**DOI:** 10.1038/s41598-022-14052-w

**Published:** 2022-06-22

**Authors:** Chung-Chih Tseng, Shi-Ying Huang, Hung-Pei Tsai, Chia-Wei Wu, Tsung-Hua Hsieh

**Affiliations:** 1 Institute of Medical Science and Technology, National Sun Yat-sen University, Kaohsiung, 80424 Taiwan; 2 Department of Dentistry, Zuoying Branch of Kaohsiung Armed Forces General Hospital, Kaohsiung, 81342 Taiwan; 3 College of Ocean Food and Biological Engineering, Jimei University, Xiamen, 361021 China; 4 Division of Neurosurgery, Department of Surgery, Kaohsiung Medical University Hospital, Kaohsiung Medical University, Kaohsiung, 80708 Taiwan; 5 Department of Medical Research, E-Da Hospital/E-Da Cancer Hospital, I-Shou University, Kaohsiung, 82445 Taiwan

**Keywords:** Cancer, Oncology

## Abstract

Oral squamous cell carcinoma (OSCC) is a common malignant tumor worldwide that is characterized by abnormal lesions or malignant hyperplasia of soft and hard tissues in the oral cavity. Previous research has found that HDAC6 may be a potential therapeutic target for cancer patients and has the ability to regulate immune cells. However, the mechanism of HDAC6 in OSCC pathogenesis is unclear. We collected clinical samples and analyzed the level of HDAC6 in OSCC patients. The results showed that in the high HDAC6 expression group, HDAC6 expression was positively correlated with the grade of OSCC (R = 0.182, *P* = 0.036) and that this group had a 3.248-fold increase in the mortality risk compared with the low HDAC6 expression group (*P* = 0.003). Survival analysis also identified a correlation between the expression of HDAC6 and overall survival in OSCC patients, and it was found that the expression of HDAC6 was inversely correlated with survival (*P* ≤ 0.001). In addition, we found that HDAC6 induced IL-13 expression through AP-1, resulting in M2 polarization of macrophages. Together, these results demonstrate that the level of HDAC6 may be a useful prognostic biomarker and offer a novel immune cell-related therapeutic strategy of targeting IL-13 in OSCC.

## Introduction

Oral squamous cell carcinoma (OSCC) is the seventh most common malignant tumor worldwide^1^ and is characterized by abnormal lesions or malignant hyperplasia of oral tissues, including the lips, the buccal mucosa (the lining of the lips and cheeks), the upper two-thirds of the tongue, the front part of the roof of the mouth (hard palate), the teeth, the sublingual floor of the mouth, the gums, and the small area behind the molars. The most common sites of OSCC in Taiwan are the tongue and buccal mucosa. OSCC mostly occurs in elderly people aged 55 to 64 years old. The main risk factors for OSCC are smoking, drinking, tobacco use, and betel nut chewing. Among these risk factors, smoking and drinking have a synergistic influence; other factors, including bad eating habits, viral infections (especially HPV infection) and poor oral hygiene, can also affect OSCC carcinogenesis^2,3^. According to cancer statistics, approximately 3% of cancers diagnosed in 2020 were OSCC, and more men than women have OSCC^1^. The most common type of OSCC is squamous cell carcinoma (SCC), which accounts for more than 90% of all OSCC cases. Other malignant tumors may arise from epithelial tissue, connective tissue, salivary glands, lymphoid tissue, and melanocytes or distant metastasis of the primary tumor^4,5^. Macrophages are multifunctional immune cells of the human innate immune system; they act through tissue-specific and environment-dependent mechanisms to regulate immune homeostasis in the body and play important roles as mediators of inflammation and antitumor immunity^6–8^. When inflammation and infection occur, blood monocytes accumulate in tissues and undergo a series of steps to differentiate into macrophages^9^. Macrophages have a high degree of plasticity, and exhibit disparate morphological manifestations under exposure to different environmental stimuli. Under physiological conditions, macrophages (M0) are induced by lipopolysaccharide (LPS), IFN-γ or TNF-α to polarize into M1-type macrophages (M1), and M1 macrophages secrete inflammatory factors (such as TNF-α, IL-1β, IL-6, and IL-12), which activate the immune system and defend against exogenous bacteria and viruses^10,11^. In contrast, when M0 macrophages are exposed to different environmental stimuli (such as IL-4 and IL-13), they polarize into M2-type macrophages (M2), which cause an anti-inflammatory response^12^. In tumors, macrophages are usually present as the M2 phenotype by exposure to environmental factors. M2 macrophages can inhibit cytotoxicity, thereby weakening their antitumor ability and promoting tumor growth^13–15^. In contrast, if the activation of M2 macrophages is blocked, the processes of tumor growth and metastasis can be inhibited, and M2 macrophages have thus been used in clinical treatment^16–19^. Current studies have shown that M1 macrophages have the ability to enhance the activation of inflammatory factors and phagocytose invading pathogens, while M2 macrophages have the opposite function, inhibiting immune functions and promoting tumor formation through IL-13. A previous study also found that an increased number of M2 macrophages correlates with a higher histopathological grade of oral squamous cell carcinoma (OSCC)^20^. However, the detailed mechanism is unclear. Interleukins (ILs) are cytokines that play an important role in the activation, differentiation, proliferation, maturation, and migration of immune cells. Interleukins constitute a large family of proteins that can promote many reactions in cells and tissues by binding to high-affinity receptors on the cell surface^21^. Among ILs, IL-13 is synthesized by CD4 + T cells (Th2), NKT cells, and mast cells, and it acts on monocytes, fibroblasts, epithelial cells, and B cells. For example, it regulates the growth and differentiation of B cells, stimulates isotype conversion to IgE (immunoglobulin E), increases the amount of mucus in epithelial cells, promotes collagen synthesis by fibroblasts, and inhibits the production of inflammatory factors^22^. Studies indicate that IL-13 is regulated by two receptors—the IL-13Ra1/IL-4 Ra complex and IL-13Ra2^23^; and IL-13Ra2 is a subunit of the IL-13R complex that has high affinity for IL-13. IL-13 induces the activity of the TGFβ1 promoter in an AP-1-dependent manner, which in turn leads to inflammation and fibrosis^24^. In addition, IL-13 transmits signals via the IL-13Rα2 and AP-1/ERK pathways to regulate tumor invasion and metastasis. According to a previous report, IL-13 regulates signal transduction through IL-13Rα2 in pancreatic cancer and ovarian cancer cells^25^. Histone deacetylase proteins (HDACs) constitute a family of enzymes that remove acetyl functional groups from histone proteins on DNA. HDAC6 has a unique structure among HDACs; it has two catalytic domains and a unique C-terminal zinc finger domain that binds to ubiquitin^26^. HDAC6 is located in the cytoplasm and mainly interacts with Non-histone proteins^27^; it is involved in many biological and pathological processes, such as cell migration, the DNA damage response and carcinogenesis, by regulating its substrates^28,29^. For example, the DNA mismatch repair protein MSH2 can be deacetylated and ubiquitinated by HDAC6 to regulate DNA mismatch repair and the DNA damage response^30^. Additionally, HDAC6 deacetylates the cell signaling regulators K-Ras and β-catenin, changing their carcinogenic activity and nuclear localization, respectively^31,32^. Related studies have shown that inhibition of HDAC6 may abolish the stability of oncogenic proteins, block tumor migration, and inhibit survival signals^33^. In addition, other studies have demonstrated that HDAC6 can regulate cancer-related signaling pathways, proving that HDAC6 has the potential to become a therapeutic target for cancer^34–36^ and has the capability to regulate immune cells. However, the understanding of the function of HDAC6 in OSCC is relatively unclear. Based on previous studies, we concluded that the tumor microenvironment, HDAC6, and IL-13 play a very important role in the carcinogenesis of OSCC. However, the correlation among them is unclear. Therefore, we hypothesized that HDAC6 is a key regulator of macrophage polarization and may be a therapeutic target to modulate IL-13. Our findings provide a scientific basis to evaluate the potential benefits of HDAC6 in human OSCC and indicate that future studies to understand the role of related molecular pathways are warranted.

## Materials and methods

### Clinical specimens and Statistical analysis

Prior to the collection of tissue samples, each subject or their legal guardian signed an informed consent form approved by the Institutional Review Board (IRB) of E-Da Hospital (EMRP-108-150). All of the study methodology satisfied the regulations issued and relevant guidelines, and approved by the IRB of E-Da Hospital. IRB provides instancedouble coded and delinked clinical specimens to us for experiments. Tissue samples were collected from 133 patients with OSCC. Detailed information on each patient, including stage, grade, tumor size, T stage, N stage, M stage, recurrence status and metastasis status, was recorded and is summarized in Table 1. Statistical analysis was performed using GraphPad Prism version 6.0 software (GraphPad, La Jolla, CA, USA). Correlations of high and low HDAC6 intensity with clinicopathologic parameters were analyzed through a univariate analysis. The significance of differences between the two independent groups was analyzed with a two-tailed Student’s t-test. *P* < 0.05 was considered to indicate a significant difference in all statistical analyses.

**Table 1:** Descriptive characteristics of the OSCC patients in the study.

Clinicopathological feature	Number of patients (N = 133)	%
**Stage**		
I/II	79	59.4
III/IV	54	40.6
**Grade**		
1	60	45.1
2	71	53.4
3	2	1.5
**Tumor size**		
> 4 cm	116	81.2
< 4 cm	17	12.8
**T stage**		
I/II	99	74.4
III/IV	34	25.6
**N stage**		
I/II	101	75.9
III/IV	32	24.1
**M stage**		
I/II	132	99.2
III/IV	1	0.8
**Recurrence status**		
Negative	102	76.7
Positive	31	23.3
**Metastasis status**		
Negative	123	92.5
Positive	10	7.5

### Immunohistochemistry

**Figure 1:**
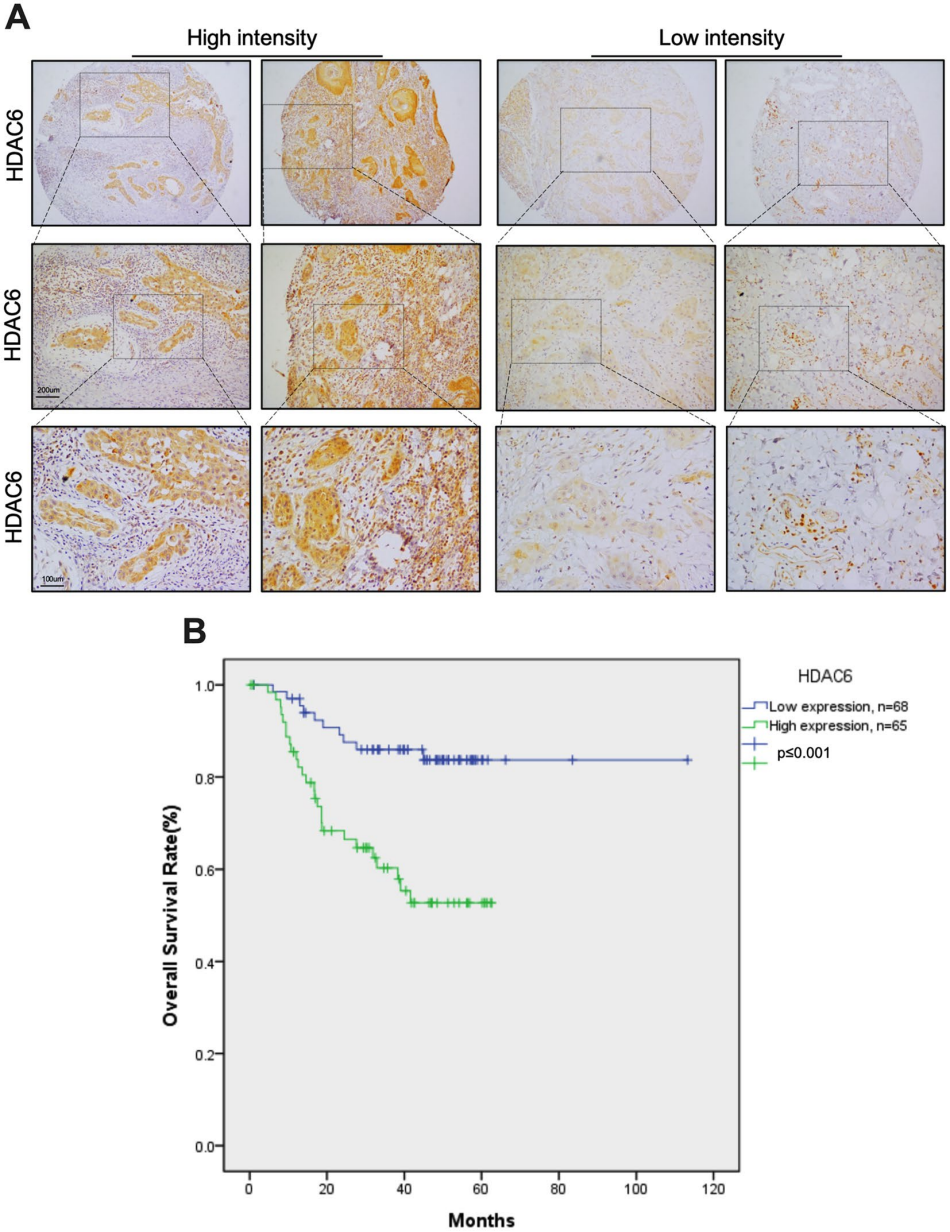
Overall survival rate of patients with OSCC stratified by HDAC6 expression. (**A**) Representative image of low and high HDAC6 intensity. (**B**) Curves showing the overall survival rates of patients with high (green line) versus low (blue line) HDAC6 levels.

Patient tissues were sliced into 4-μm sections and stained with an automated Bond-Max immunostainer following the manufacturer’s protocol (Leica Microsystems). Slides were immunostained with a human anti-HDAC6 (Santa Cruz, sc-28386) antibody and counterstained with Mayer's hematoxylin (Sigma). Immunohistochemistry staining was assessed by 2 pathologist and clearly define for high and low intensity. The staining interpretation method was based on Iliopoulos D et al., 2005^37^. The total score is calculated by multiplying the tumor cell range by the intensity. Representative images of HDAC6-positive and -negative tissues are presented in Fig. 1A.

### Cell lines

The human oral squamous cell carcinoma (OSCC) cell lines OC-2, OECM-1 and human monocytic cell line (THP-1) were obtained from the American Type Culture Collection (ATCC) and maintained in DMEM/F12 culture medium (Gibco, France) supplemented with 1% penicillin/streptomycin and 10% fetal bovine serum (FBS) (Gibco) in a humidified incubator at 37 °C and 5% CO2.

### Transfection and Luciferase assay

Cells (1X105) were seeded in 6-well plate. After 24 h, 2 µg of HDAC6 plasmid was transfected with TurboFect in vivo Transfection Reagent following the manufacturer’s protocol. For the luciferase assay, an IL-13 promoter sequence fragment (CTGGGAGTCAGAG) was synthesized and cloned into the pGL3-Basic expression vector (Promega). Luciferase activities were measured with a Dual-Luciferase Reporter Assay system and a GloMax 20/20 luminometer (Promega). In addition, luciferase assay data are presented as the mean ± SD of three independent replicates. *P* < 0.05 was considered to indicate a significant difference in all statistical analyses.

### Cell growth assay

The cell growth assay was analyzed by Cell Counting Kit-8(CCK-8, Dojindo, Kumamoto, Japan). Brief, HDAC6-overexpring (5 × 10^3^) cell were seeded into a 96-well plate 24 h before experimentation. Transient transgene expression occurs within 2–72 h after transfection according to TurboFect in vivo Transfection Reagent manufacturer’s protocol. Therefore, we monitoring the cell growth for 24, 48 and 72 h after HDAC6-overexpressing. The absorbances of each sample were measured at 450 nm in Microplate Reader.

### Immunoblot analysis

Lysed cells were extracted with RIPA Cell Lysis Buffer, and proteins were transferred onto Amersham Hybond 0.45 PVDF membranes (GE Healthcare). The blots was cut prior and hybridization with antibodies. The following antibodies used for immunoblotting were purchased: anti-HDAC6(Santa Cruz, sc-28386), anti-IL13(Sigma, 3596M1), anti-p-AP1(Biovision, A1249), anti-AP1(Biovision, #3009) and anti-β-actin (Sigma, SAB5600204). The quantify of western blot bands was analyzed by Image J software in the Supplemental Materials.

### RNA extraction and qPCR

To confirm that HDAC6 regulates the polarization of macrophages, the mRNA levels of polarization genes, including M1 (iNOS and TNFα) and M2 (IL-10 and arginase I) polarization genes, were determined by reverse transcription-PCR (RT-PCR) and quantitative PCR (qPCR). Cellular RNA was extracted by TRIzol (Invitrogen) and transcribed into cDNA with a Reverse Transcription System Kit (Promega, Madison, WI). The primers (Genemessenger Scientific, Tainan, Taiwan) used in the experiment were as follows. iNOS: forward, 5′-CAG-CTG-GGC-TGT-ACA-AAC-CTT-3′ and reverse, 5′-CAT-TGG-AAG-TGA-AGC-GTT-TCG-3′. TNF-α: forward, 5′-AAA-ATT-CGA-GTG-ACA-AGC-CTG-TAG-3′ and reverse, 5′-CCC-TTG-AAG-AGA-ACC-TGG-GAG-TAG-3′. IL-10: forward, 5′-AGA-AGC-ATG-GCC-CAG-AAA-TC-3′ and reverse, 5′-CCA AGG-AGT-TGT-TTC-CGT-TAGC-3′; Arginase 1: forward, 5′-AGA-GCT-GAC-AGC-AAC-CCT-GT-3′ and reverse, 5′-GGA-TCC-AGA-AGG-TGA-TGG-AA-3′. QPCR was performed with SYBR Green PCR Master Mix (Applied Biosystems, Foster City, CA) in an ABI 7500 Real-Time PCR System (Applied Biosystems).

## Results

### Correlation between the HDAC6 expression profile and unfavorable clinicopathological parameters

To confirm whether the expression of HDAC6 is related to the prognosis and survival of OSCC patients, we initially collected clinical samples from 133 OSCC patients and recorded the patients’ clinical characteristics, including stage, grade, tumor size, T stage, N stage, M stage, recurrence status and metastasis status. We used the descriptive statistical analysis mode in SPSS statistical software and found that most of the patients had grade 1 (45.1%) or grade 2 (53.4%) OSCC (Table 1). Next, we analyzed HDAC6 expression in the clinical specimens of 133 OSCC patients and performed immunohistochemical staining on these specimens using an automatic staining machine. We classified patients into groups with high and low intensity (Fig. 1A) with the assistance of a pathologist and performed statistical analysis of the correlation between HDAC6 expression and the clinical features of OSCC. We found that the expression of HDAC6 was positively correlated with the grade of OSCC (R = 0.182) and that the correlation was statistically significant (*P* < 0.05) (Table 2). Furthermore, we used linear regression (Cox) analysis to evaluate correlations with clinicopathological features. The results showed that the mortality risk in the high HDAC6 expression group was 3.248-fold greater than that in the low HDAC6 expression group and that this difference was statistically significant (*P* = 0.003) (Table 3).

**Table 2:** Relationship between the HDAC6 intensity level and clinicopathologic parameters of patients with OSCC.

Clinicopathological parameter	HDAC 6 Intensity			
	Low	High	R	*P*-value
**Stage**			− 0.12	0.891
I/II	40	39		
III/IV	28	26		
**Grade**			0.182	0.036
1	36	24		
2	32	39		
3	0	2		
**Tumor size**			− 0.14%	0.874
> 4 cm	59	57		
< 4 cm	9	8		
**T stage**			− 0.21	0.808
I/II	50	49		
III/IV	18	16		
**N stage**			0.83	0.342
I/II	54	47		
III/IV	14	18		
**M stage**			− 0.85	0.33
I/II	67	65		
III/IV	1	0		
**Recurrence status**			0.101	0.246
Negative	55	47		
Positive	13	18		
**Metastasis status**			0.121	0.167
Negative	65	58		
Positive	3	7

**Table 3:** Multivariate Cox regression analysis of overall survival for patients with OSCC.

Clinicopathological parameter	Odds Ratio (OR)	95% CI		
		Lower	Upper	*P*-value
HDAC6	3.248	1.488	7.091	0.003
Stage	1.073	0.275	4.189	0.919
Grade	1.067	0.49	2.322	0.87
Tumor size	0.936	0.278	3.151	0.915
T stage	2.435	0.782	7.585	0.125
N stage	2.604	0.89	7.616	0.081
Recurrence status	1.615	0.715	3.647	0.249
Metastasis status	4.514	1.752	11.627	0.002

*CI* confidence interval.

Finally, we analyzed the relationship between the expression of HDAC6 and the overall survival rate of OSCC patients through Kaplan–Meier analysis, and the results showed that the expression of HDAC6 was inversely correlated with the survival rate. When the expression of HDAC6 was high, the survival rate of patients with OSCC was decreased, and when the expression of HDAC6 was low, the survival rate was increased (Fig. 1B,  *P* ≤ 0.001). The expression level of HDAC6 was thus correlated with the survival of OSCC patients.

### HDAC6 promotes cell growth in OC-2 and OECM-1 cell lines

**Figure 2:**
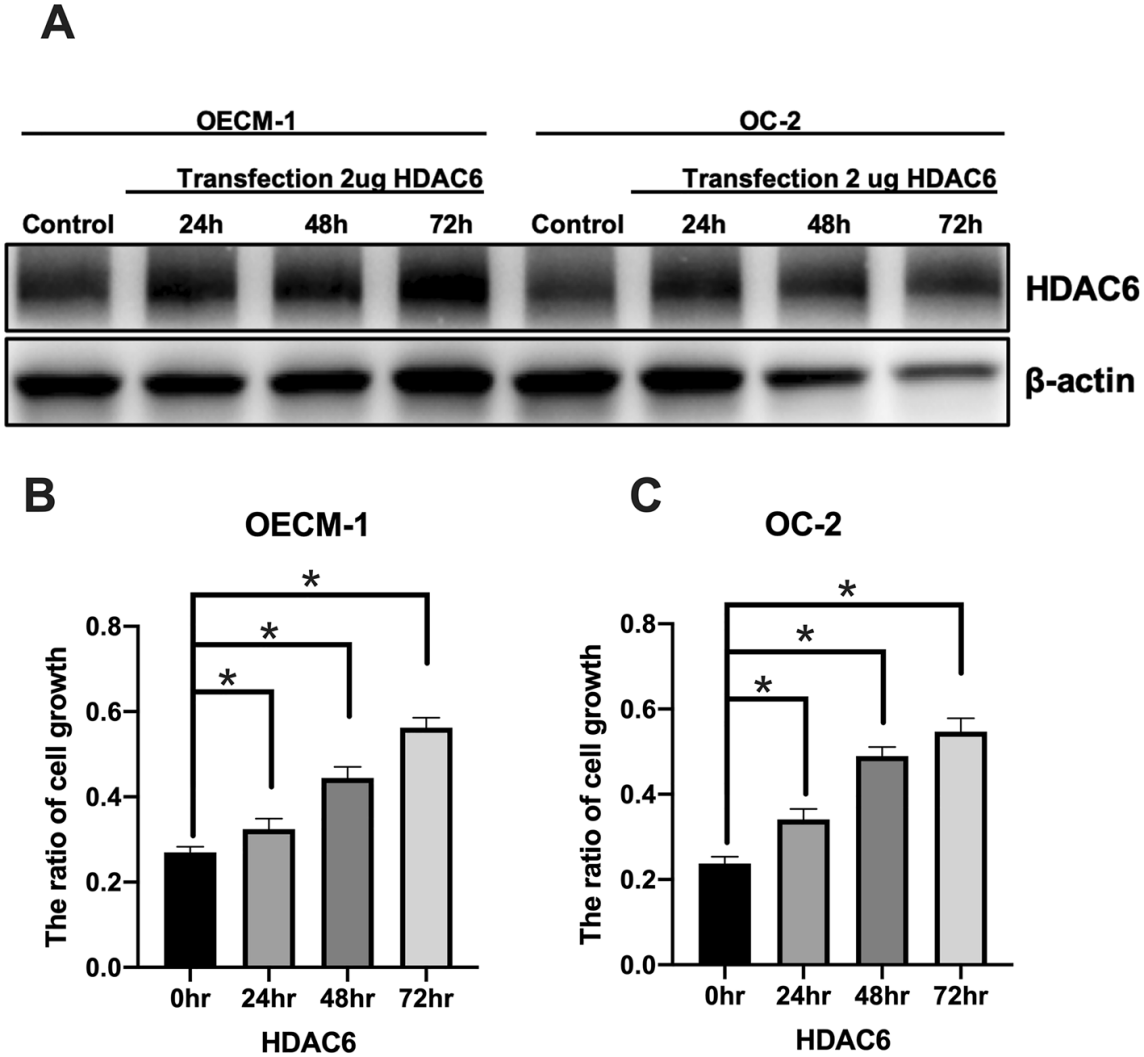
HDAC6 mediates the growth of OSCC cells. The HDAC6 plasmid (2 µg) was transfected into cell lines. After 24, 48 and 72 h, (**A**) the level of HDAC6 was analyzed by western blotting, and (**B** and **C**) cell growth was analyzed by a CCK-8 assay.

To analyze the effect of HDAC6 on cell growth in oral cancer cell lines, we evaluated cell growth with a CCK-8 assay in the OC-2 and OECM-1 cell lines. We transfected these cells with the HDAC6 overexpression plasmid, and the protein level of HDAC6 was analyzed by western blotting (Fig. 2A). The results showed that HDAC6 promoted cell growth in the OC-2 and OECM-1 cell lines (Fig. 2B and C).

### HDAC6 induced the expression of IL-13 through AP-1

**Figure 3:**
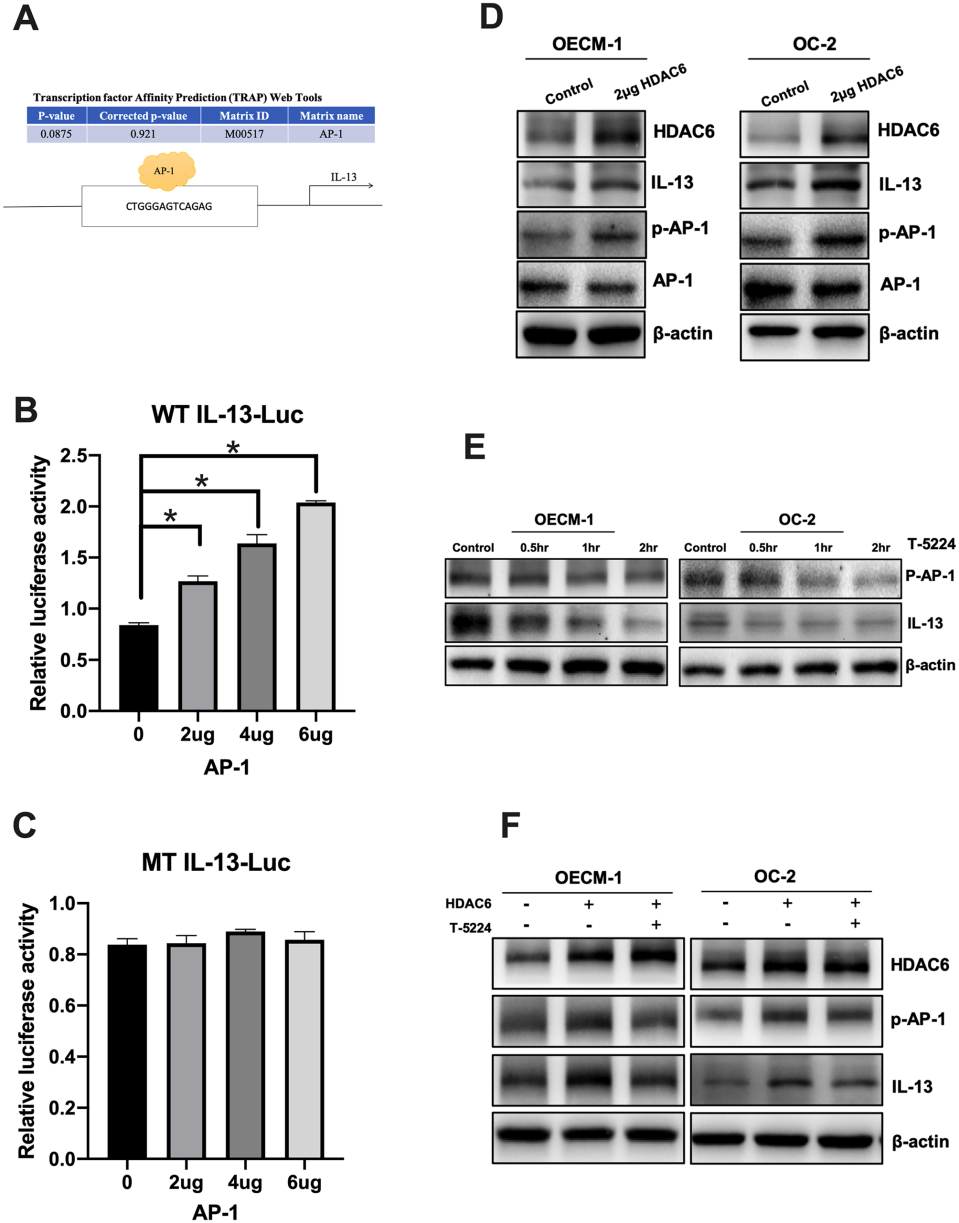
HDAC6 induced IL-13 expression via AP-1. (**A**) Transcription factor affinity prediction (TRAP) software predicted that the promoter region of IL-13 has an AP-1 transcription binding sequence. (**B**, **C**) pGL3-IL-13 luciferase plasmids containing wild-type (WT IL-13-Luc) or mutated (MT IL-13-Luc) 5′-UTRs were constructed. 293 T cells were cotransfected with the IL-13 5′-UTR plasmids and a plasmid expressing AP-1 at different dose ratios. (**D**) The HDAC6 plasmid (2 µg) was transfected into OECM-1 cells, and OC-2, HDAC6, IL-13, AP-1, p-AP-1 and B-actin were analyzed by western blotting. B-actin was used to normalize protein expression levels. (**E**) The AP-1 inhibitor T-5224 (40 µM) and (F) 2 µg of the HDAC6 plasmid were transfected into cells, and the level of IL-13 was analyzed by western blotting.

We next analyzed the ability of HDAC6 to regulate macrophage polarization into alternatively activated (M2) macrophages. A previous study found that IL-13 induced macrophage polarization from the M0 phenotype to the M2 phenotype^38^. In addition, treatment with an HDAC6 inhibitor was found to decrease the production of the Th2 cytokine Il-13^39^. Therefore, we speculated that HDAC6 may have the ability to induce the expression of IL-13. Transcription factor affinity prediction (TRAP) software was used to predict the transcription factor binding sites, and a binding motif for AP-1 (TGAGTCA) was identified in the regulatory region of the IL-13 promoter (Fig. 3A).To test the abovementioned hypothesis, luciferase reporter plasmids (pGL3) encoding wild-type (WT) and mutated (MT) fragments of the IL-13 5′-UTR were constructed and cotransfected with an AP-1 plasmid into HEK-293 T cells. We found that AP-1 overexpression increased luciferase reporter activity in a dose-dependent manner (Fig. 3B) in the WT group but not the mutated group (Fig. 3C). Next, to investigate the expression of IL-13, the OSCC cell lines OC-2 and OECM-1 were transfected with the HDAC6 overexpression plasmid, and the expression of IL-13 was determined by western blotting. The results showed that HDAC6 induced the expression of IL-13 in the OC-2 and OECM-1 cell lines (Fig. 3D). In addition, we used an AP-1 inhibitor (T-5224) to block the mechanism of AP-1, and these results showed that AP-1 phosphorylation was activated by HDAC6 overexpression but blocked by treatment with the AP-1 inhibitor (T-5224) in OSCC cell lines (Fig. 3E, F).

### HDAC6 mediated THP-1 macrophage polarization into alternatively activated (M2) macrophages

**Figure 4:**
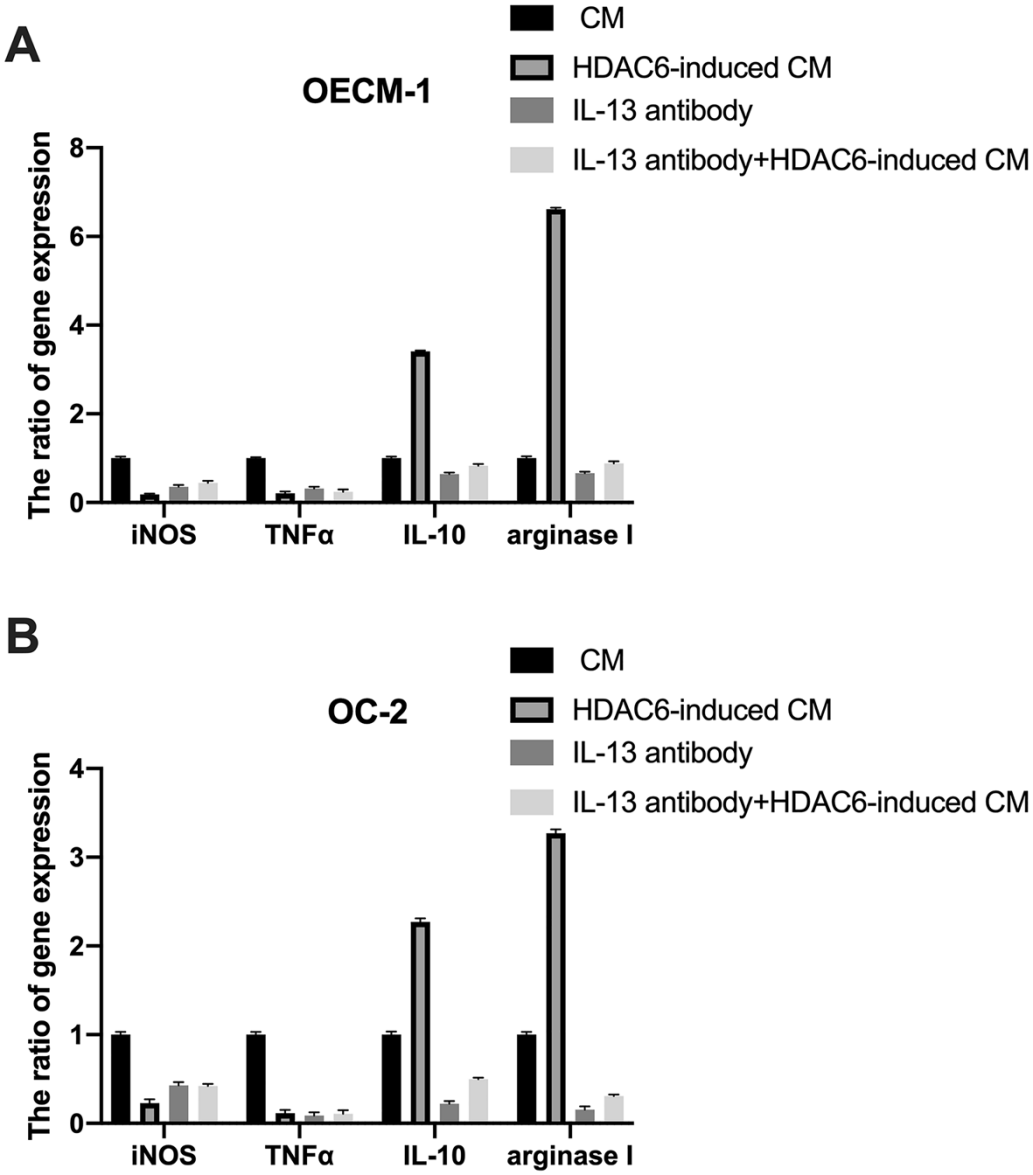
HDAC6-induced CM promoted M1 polarization of macrophages. CM, HDAC6-induced CM, IL-13 antibody and IL-13 antibody + HDAC6-induced CM from OEC-M1 (**A**) or OC-2 (**B**) cells was used to treat THP-1 cells. After 24 h, THP-1 cell mRNA was extracted, and iNOS, TNFα, IL-10 and arginase I gene expression was analyzed by qPCR.

To explore whether HDAC6 can regulate the polarization of macrophages, we collected the supernatant of OSCC cells overexpressing HDAC6. The supernatant from untreated cells was called conditioned medium (CM), and the supernatant from the HDAC6-overexpressing cells was called HDAC6-induced CM. The mRNA levels of the M1 (iNOS and TNFα) and M2 (IL-10 and arginase I) macrophage markers were analyzed by qPCR after THP-1 cells were exposed to CM, HDAC6-induced CM, IL-13 antibody and IL-13 antibody + HDAC6-induced CM. We found that compared to CM, HDAC6-induced CM from THP-1 cells significantly increased the expression of IL-10 and arginase I, but blocked by IL-13 antibody. (Fig. 4A,B). Therefore, we hypothesized that HDAC6 activates M2 polarization of THP-1 macrophages through the AP-1/IL-13 signaling pathway.

## Discussion

Many previous studies have found that macrophages have both positive and negative effects on tumor growth in various cancers. The ratio of M1 to M2 macrophages plays a major regulatory role and affects the function of the immune system. It is currently known that an increase in the number of M2 macrophages is related to the developmental process of oral squamous cell carcinoma^40^, to angiogenesis and to more malignant histopathological features (i.e., higher grade)^41^. Our study found that HDAC6 expression was significantly associated with the tumor grade and mortality risk in OSCC. Therefore, HDAC6 expression and macrophage polarization are strongly correlated. According to our results, HDAC6 induced IL-13 through AP-1 and activated the polarization of M0 macrophages into M2 macrophages. In addition, silencing HDAC6 decreased macrophage adhesion to the extracellular matrix and significantly blocked LPS-induced activation of macrophages. Hyperacetylation of tubulin in microtubules may impair microtubule dynamics, leading to failure of LPS-induced M1 activation^42^. We also found that HDAC inhibition shifted macrophage polarization toward the M2 phenotype through the GSK3β/PTEN/Akt axis^43^. Therefore, we believe that HDAC6 has the ability to regulate M1/M2 polarization of macrophages. The transcription factor activator protein-1 (AP-1) is a downstream factor of the M-CSF/MAPK signaling pathway and is a major transcription factor that mediates proinflammatory and activated macrophages^44,45^. Some results indicate that IL-6 mediates OSCC cell migration through the SYK/JNK/AP-1 signaling pathway^46^. Previous studies have found that HDAC6 induces the expression of proinflammatory genes (such as IL-1β, IL-6 and TNF-α) via the ROS-MAPK-NF-κB/AP-1 signaling pathway in macrophages^47^. TNF-α, IL-1β, IL-6, and IL-12 are secreted from M1 macrophages and activate the immune system to defend against exogenous bacteria and viruses. Interestingly, another study also found that the production of the Th2 cytokine IL-13 was induced by AP-1^25^ and that selective HDAC6 inhibition impaired IL-13 expression^39^. A previous study found that the salivary level of IL-13 was significantly different in OSCC patients compared to controls and that IL-13 could prove to be a potential biomarker for OSCC^48^. This finding is consistent with our experimental results showing that HDAC6 induced M2 polarization through the AP-1/IL-13 signaling pathway. Therefore, AP-1-mediated regulation of macrophage polarization still needs further in-depth study and confirmation of its true role.

## Supplementary Information

Supplementary Figures.

## Data Availability

The datasets generated during the current study are available from the corresponding authors on reasonable request.
