# Supplementary material for: HDAC6 is a prognostic biomarker that mediates IL-13 expression to regulate macrophage polarization through AP-1 in oral squamous cell carcinoma
Source: Sci Rep. 2022 Jun 22;12:10513. doi: 10.1038/s41598-022-14052-w (PMC9217956; doi:10.1038/s41598-022-14052-w)

**HDAC6 is a prognostic biomarker that mediates IL-13 expression to regulate macrophage polarization through AP-1 in oral squamous cell carcinoma**

Chung-Chih Tseng^1,2^, Shi-Ying Huang^3^, Hung-Pei Tsai^4^, Chia-Wei Wu^5^ and Tsung-Hua Hsieh^5*^

^1^ Department of Dentistry, Zuoying Branch of Kaohsiung Armed Forces General Hospital, Kaohsiung, 81342, Taiwan

^2^ Institute of Medical Science and Technology, National Sun Yat-sen University, Kaohsiung, 80424, Taiwan

^3^ College of Food and Biological Engineering, Jimei University, Xiamen 361021, China;

^4^ Department of Neurosurgery, Kaohsiung Medical University Hospital, Kaohsiung Medical University, Kaohsiung, 80708, Taiwan;

^5^ Department of Medical Research, E-Da Hospital/E-Da Cancer Hospital, I-Shou University, Kaohsiung, 82445, Taiwan;

*Correspondence: Tsung-Hua Hsieh, PhD, Tel.: 886-7-6151100 ext 5072, Fax: 886-7-311-2493, E-mail address: pelagice@yahoo.com.tw, Mailing address: 6 Yi-Da Rd., Yan-Chao District, Kaohsiung 82445, Taiwan

### The blots was cut prior and hybridization with antibodies.

### Full-length gels and blots for 2A

###
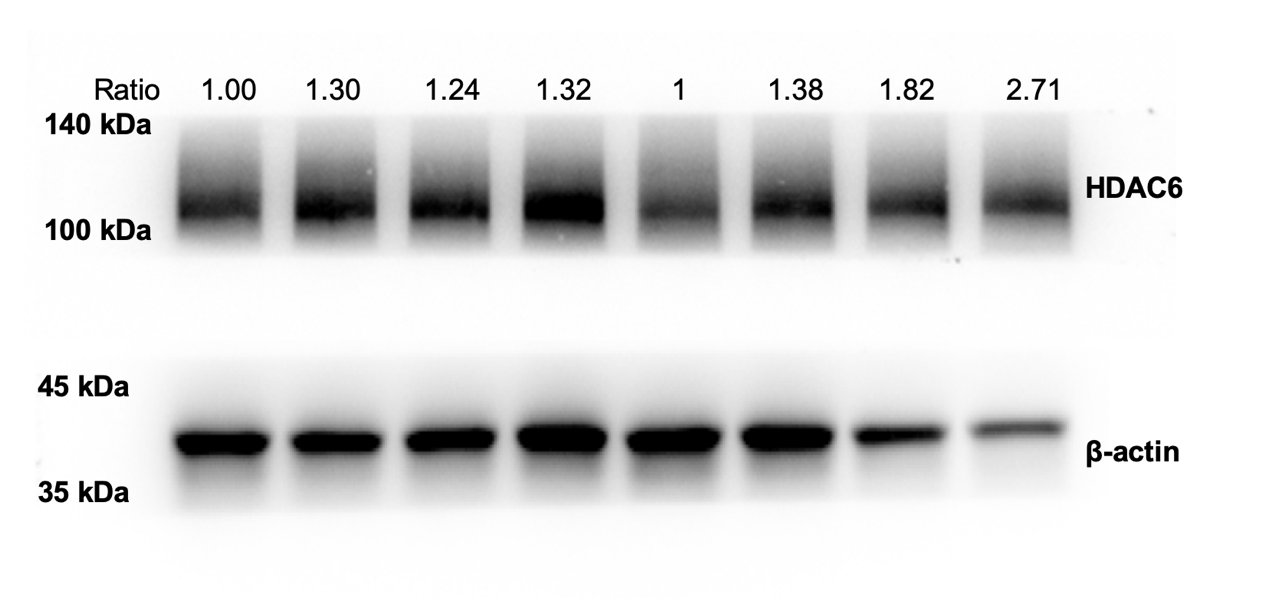


### Full-length gels and blots for 3D, E and F

###
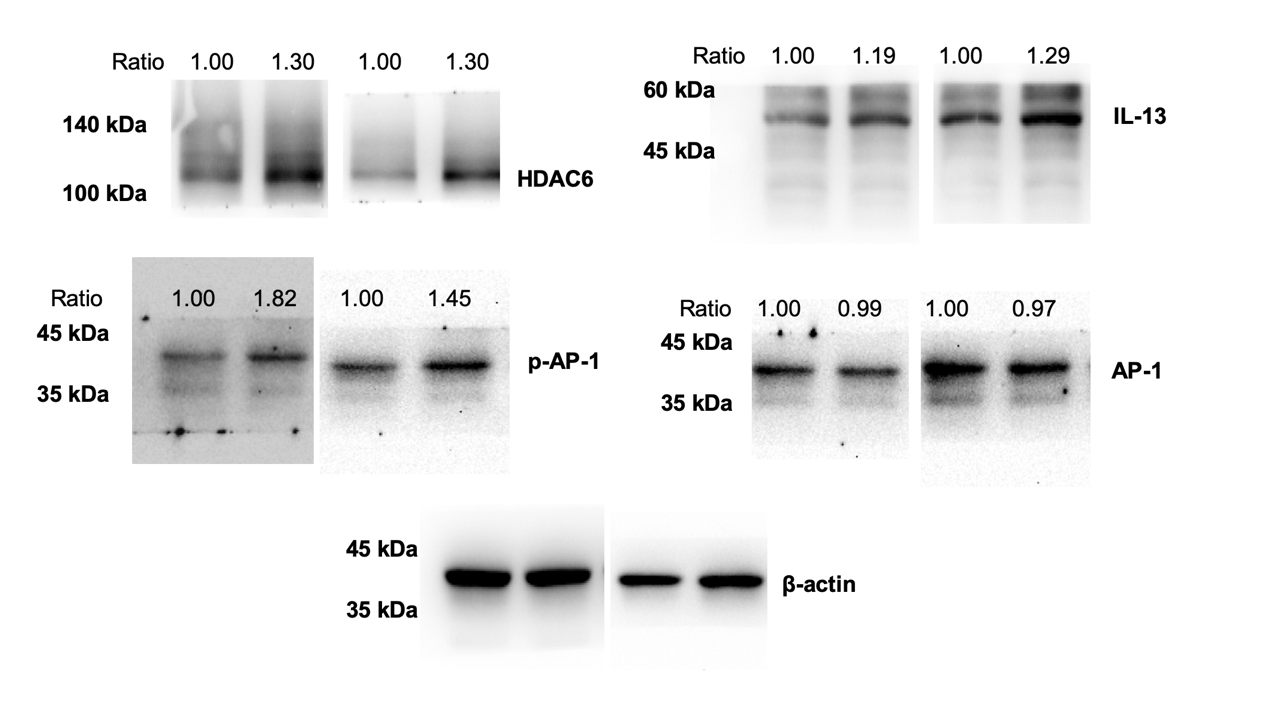


###
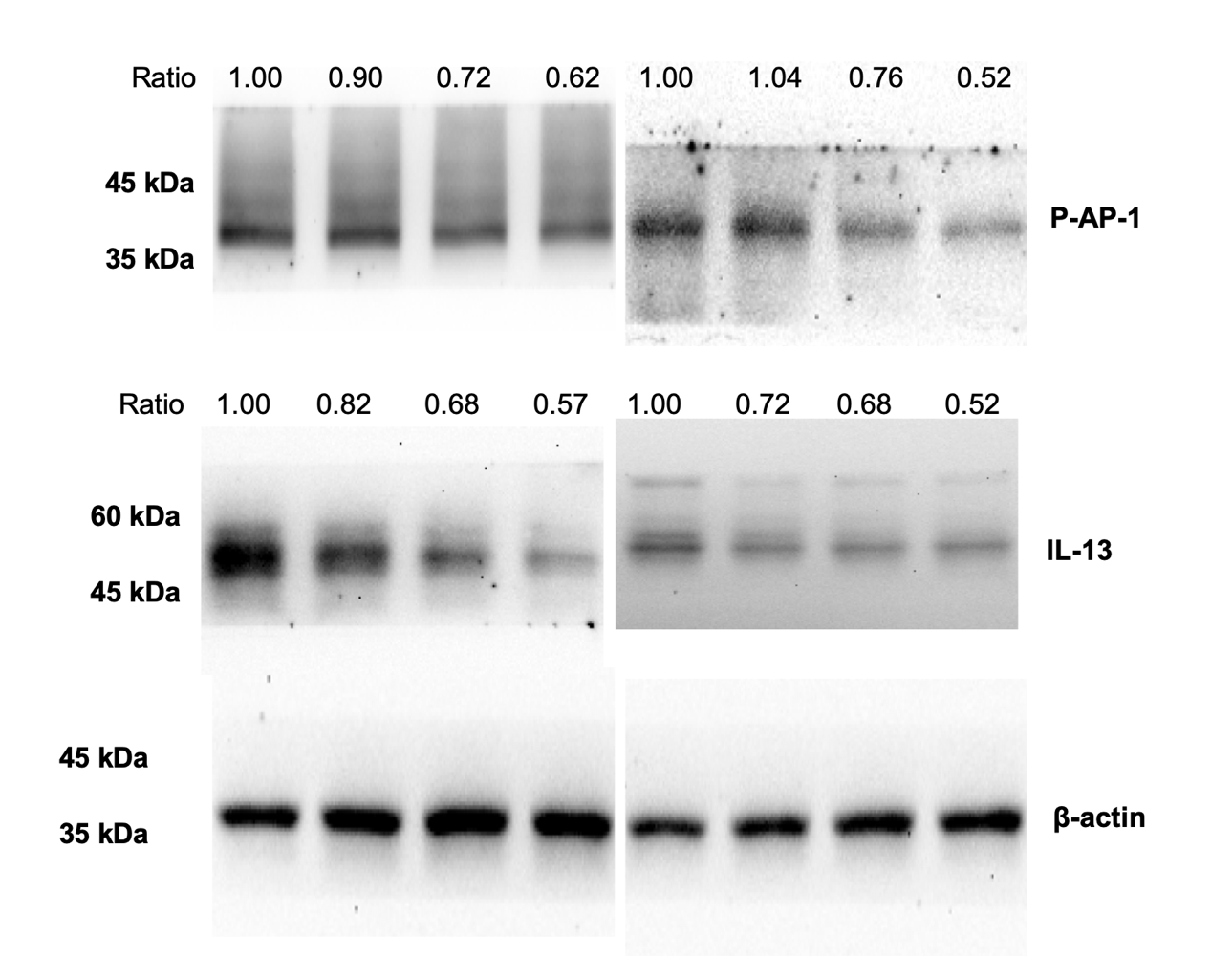


###
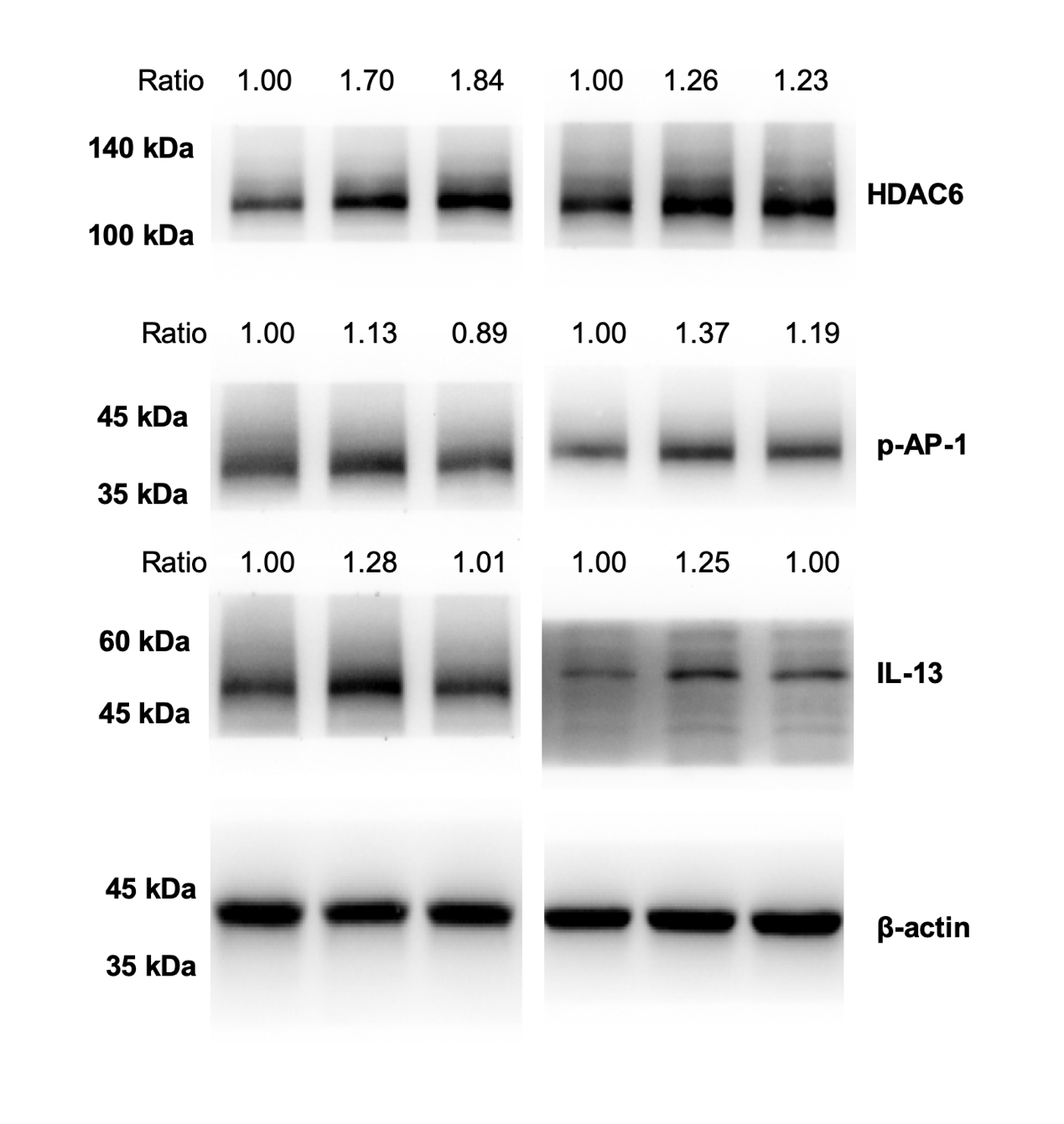

Supplement: Supplementary file 1 — Supplementary Figures. [file 41598_2022_14052_MOESM1_ESM.docx]
